# Ecological Processes Affecting Long-Term Eukaryote and Prokaryote Biofilm Persistence in Nitrogen Removal from Sewage

**DOI:** 10.3390/genes11040449

**Published:** 2020-04-20

**Authors:** Inga Leena Angell, Linda Bergaust, Jon Fredrik Hanssen, Else Marie Aasen, Knut Rudi

**Affiliations:** Faculty of Chemistry, Biotechnology and Food Science, Norwegian University of Life Sciences, 1430 Ås, Norway; inga.angell@nmbu.no (I.L.A.); linda.bergaust@nmbu.no (L.B.); jon.hanssen@nmbu.no (J.F.H.); else-marie.aasen@nmbu.no (E.M.A.)

**Keywords:** biofilm, stability, sewage treatment, microbiome

## Abstract

The factors affecting long-term biofilm stability in sewage treatment remain largely unexplored. We therefore analyzed moving bed bioreactors (MBBRs) biofilm composition and function two years apart from four reactors in a nitrogen-removal sewage treatment plant. Multivariate ANOVA revealed a similar prokaryote microbiota composition on biofilm carriers from the same reactors, where reactor explained 84.6% of the variance, and year only explained 1.5%. Eukaryotes showed a less similar composition with reactor explaining 56.8% of the variance and year 9.4%. Downstream effects were also more pronounced for eukaryotes than prokaryotes. For prokaryotes, carbon source emerged as a potential factor for deterministic assembly. In the two reactors with methanol as a carbon source, the bacterial genus *Methylotenera* dominated, with *M. versatilis* as the most abundant species. *M. versatilis* showed large lineage diversity. The lineages mainly differed with respect to potential terminal electron acceptor usage (nitrogen oxides and oxygen). Searches in the Sequence Read Archive (SRA) database indicate a global distribution of the *M. versatilis* strains, with methane-containing sediments as the main habitat. Taken together, our results support long-term prokaryote biofilm persistence, while eukaryotes were less persistent.

## 1. Introduction

Micro-organisms organized in the form of biofilms represent one of the most successful forms of life on Earth [1]. Their functional stability and efficiency make them ideal biological engines for application in wastewater treatment, such as in nitrogen (N) removal [2,3]. However, we lack understanding of biofilm processes in wastewater treatment. In particular, we do not know the relative contributions of stochastic and deterministic processes in long-term biofilm assembly [4]. Moving bed bioreactor (MBBR) systems are extensively used in cold water wastewater treatment processes due to their large area for biofilm formation, where homogenous plastic-based biofilm carriers in suspension support biofilm growth [5]. MBBR-based systems have therefore been used to address questions about biofilm thickness [6,7], potential effect of predation [8] and the effect of fluctuations in temperature and nutrients. Ecological processes affecting long-term biofilm persistence, however, have not yet been addressed [9], despite the importance of biofilms in nature [10].

Prokaryote community assembly has traditionally been considered deterministic due to the small size and high numbers of prokaryotes [11], whereas eukaryote community assembly has been considered more stochastic due to the larger size and lower numbers of eukaryotes [12,13]. Recently, stochastic processes have also been proposed as important for prokaryotes [14]. For wastewater systems, the turnover of heterotrophic bacteria are better explained by stochastic rather than deterministic models [15]. Furthermore, in systems with one-way liquid flow, increased downstream diversity has been inferred as a consequence of stochastic processes due to detachment of upstream prokaryotes [16].

The aim of our work was to address assembly processes of biofilms through analyzing both the prokaryote and eukaryote microbiota composition and function two years apart from a MBBR sewage treatment plant for nitrogen (N) removal in Norway. This was done by a combination of functional, taxonomic (both prokaryote and eukaryote), proteomic and metagenomic analyses of individual biobead microbiota, in addition to investigation of global distributions through Sequence Read Archive (SRA) searches [17].

**Figure 1:**
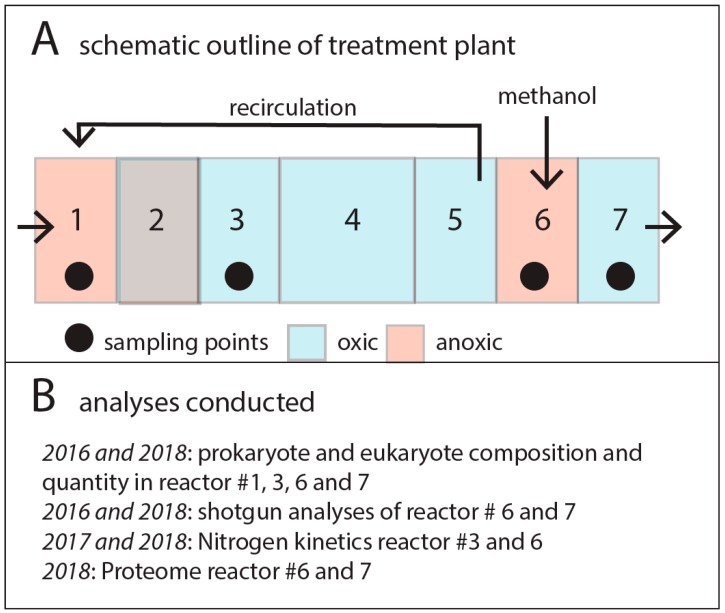
Schematic outline of (**A**) the nitrogen-removal plant, and (**B**) the analyses conducted. (**A**) The plant consisted of 7 reactors, where Reactor #2 was switched between oxic and anoxic conditions (oxic during winter and anoxic during summer). (**B**) Summary of the analyses conducted for the different years and reactors. Samplings for both 2016 and 2018 were done in March.

N-removal MBBR systems combine sewage reflux with different environmental conditions in a chain of reactors. A typical approach for N removal is to utilize the innate nitrification and denitrification capabilities of the wastewater microbiota. Nitrification is the oxygen-dependent oxidation of ammonia/ammonium (NH_3_/NH_4_^+^) to nitrite (NO_2_^−^), followed by oxidation of nitrite to nitrate (NO_3_^−^). Denitrification is the reduction of nitrate to N_2_ via nitrite, NO and N_2_O, and is performed by facultative aerobes when oxygen is scarce. It is a highly energy-yielding process, second only to oxygen respiration, and the trait is widespread across phyla [18]. Through manipulation of, for example, oxygen availability and carbon content, the microbial community may be pushed towards nitrification or denitrification, allowing the removal of NH_4_^+^/NH_3_ and NO_3_^−^/NO_2_^−^ from wastewater. Ideally, this should take place with minimal release of intermediates such as NO or N_2_O, due to the environmental effects of these nitrogenous gases [19,20]. An outline of the process for the plant analyzed in our work is given in Figure 1A.

## 2. Materials and Methods

### 2.1. Experimental Site and Setup

The Nordre Follo sewage treatment plant that was analyzed in this work covers the densely populated regions Ski and Ås south of Oslo, Norway. The plant processes 4–5 million m^3^ of sewage annually, containing about 150 tons of nitrogen, with Oslofjord as the recipient. The plant includes a biological nitrogen-removal stage with removal of about 80% of the nitrogen due to the vulnerability of the Oslofjord. The biological nitrogen-removal system consists of 7 reactors containing a total of 1 billion Kaldnes K1 Bio Media (Kruger Kaldnes, Oslo, Norway) with a diameter of 10 mm and height of 7 mm for biofilm formation. Reactor #1 is denitrifying using sewage as a carbon source. Reactors #2 to 5 are nitrifying with a reflux to Reactor #1. Reactor #6 is denitrifying with methanol as a carbon source, and finally Reactor #7 is included to respire traces of methanol before outlet in Oslofjord at 50 m depth.

An outline of the experimental setup and analytical approaches is provided in Figure 1B. The reactors analyzed were chosen to cover all the N-transformation reactions. To address microbiota persistence, we reanalyzed the reactors in 2018, exactly 2 years after our initial analyses in 2016 [8].

### 2.2. DNA Extraction

Sampling and DNA extraction were carried out as previously described [8], where each sample corresponded to one individual biobead. In addition to sampling in March 2016 (*n* = 36; 9 from each of Reactors #1, #3, #6 and #7), a second sampling was carried out in March 2018 (*n* = 36; 9 from each of Reactors #1, #3, #6 and #7), resulting in a total number of 72 samples.

### 2.3. Amplicon Sequencing

A two-step PCR amplification was carried out as previously described [8], using the primer pairs PRK341F/PRK806R [21] targeting the V3–V4 region of the prokaryotic *SSU* gene, and 3NDF /V4EukR2 [22] targeting the V4 region of the eukaryotic *SSU* gene. Sequencing was performed on a MiSeq platform (Illumina, CA, USA) using a V3 chemistry kit for 300 bp paired-end reads. Resulting reads were joined and demultiplexed before quality filtering where singletons were removed; min length was set to 350 and maxEE = 1.0. Clustering was done using USEARCH 8.0 [23] with ≥ 97% similarity. Taxonomy was assigned using UCLUST and the SILVA 128 database [24]. Diversity analysis was performed using QIIME [25], with a sequencing depth of 10,000 prokaryotic and 1000 eukaryotic sequences per sample. Diversity analyses included Simpson and Shannon α-diversity indexes, in addition to β-diversity analyses using the Bray–Curtis distances. For prokaryotes, 66 samples passed the rarefaction criteria, while for eukaryotes, 36 passed the criteria.

Functional assignments for prokaryotes was done through matches with the MIDAS 2.0 database [26] in May 2018.

### 2.4. Whole Metagenome Sequencing

Four samples from both 2016 and 2018 (Reactor #6 *n* = 2, Reactor #7 *n* = 2) were chosen for whole genome shotgun sequencing. Preparation of the sequencing library was carried out using the Nextera XT kit (Illumina, CA, USA) as recommended by the supplier, and sequencing was performed on a MiSeq platform using a V3 chemistry kit for 300 bp paired-end reads. The reads were then assembled using the microbial metagenomic toolbox in CLC genomic workbench (Qiagen, Hilden, Germany), before binning and annotation in the PATRIC database [27].

### 2.5. SRA Database Search

We used the recently developed SRA search tool [17] to identify similar sequences among the available raw sequence data from metagenomic samples in the NCBI Sequence Read Archive (SRA). The search tool uses PARTIE [28] to separate metagenomic and amplicon sequences, bowtie2 for read alignment [29] and the computing resources of XSEDE [30].

The reads were filtered in order to ensure that hits for complete sequence elements were high accuracy read hits (mapping quality score > 38), with at least 200 read hits covering the whole sequence elements (with hits within 10,000 bp of both ends of the query sequence). Final filtering was done in the MATLAB programming environment.

### 2.6. Proteome Analysis

A proteome analysis was conducted on samples from each of Reactors #6 and #7 in 2018, but not on the samples from 2016. Pellet from biobead was resuspended in 500 uL 4% SDS, 50 mM Tris and 10 mM DTT before a heat treatment at 95 °C for 10 min and processed at 1800 rpm for 45 s × 3 in FastPrep96 (MP Biomedicals, CA, USA) using 500 uL of acid-washed glass beads (<106 um, Sigma-Aldrich, Hamburg, Germany). The samples were then centrifuged at 13,000 rpm for 5 min at 4 °C and the supernatant was stored at −20 °C until ready to use. The sample was further processed using the Strap method as previously described [31], before LC-MS/MST separation on an Ultimate 3000 RSLCnano-Qexactive (Thermo Scientific, USA). The resulting data were analyzed using Scaffold 4 (Proteome Software, OR, USA) using annotations from the shotgun sequence assemblies as a database.

### 2.7. Nitrogen Turnover in Biobeads from Reactors #3 and #6

Nitrification and denitrification activity were monitored in biobeads sampled in March 2017 and 2018 from Reactors #3 (nitrification) and #6 (denitrification), respectively. These analyses were not conducted in 2016. Biobeads were transferred to 120 mL serum vials containing 50 mL liquid medium and magnetic stirring bars. Each vial contained 20 biobeads in liquid form from the respective reactors. Vials with biobeads and liquid from Reactor #3 were supplemented with NH_4_Cl to a final concentration of 1 mM. Reactor #6 vials contained 1/10 TSB medium (C/N-substrate) and 10 mM KNO_3_ (terminal electron acceptor, denitrification). These were sealed with rubber septa and the headspace atmosphere was replaced by helium through 6 repeated cycles of evacuation and He filling. Anoxic vials (*n* = 6) were then placed at 15 °C in a semi-automatic incubation system with room for 15 stirred vials. The incubation system consisted of a thermostatic water bath with a magnetic stirring plate and an autosampler connected to a micro GC and a NO analyzer [32]. This allowed frequent sampling from headspace and quantification of O_2_, CO_2_, NO, N_2_O and N_2,_ and thus high-resolution monitoring of the intermediates and products of denitrification. Vials with liquid and biobeads from Reactor #3 were incubated at 15 °C under aerobic conditions. However, representative vials (*n* = 4) were sealed with rubber septa and aluminum caps and monitored in the incubation system to assess the release of N_2_O and NO during nitrification. Liquid samples were collected at T = 0 and at endpoint (T = 15 h and 36 h for denitrification and nitrification vials, respectively) and from unamended reactor water. Debris was removed by centrifugation and supernatants stored at −20 °C pending analyses. NH_4_^+^/NH_3_, NO_2_^−^ and NO_3_^−^ were quantified in liquid samples using standard colorimetric assays: ortho-phthaldialdehyde, the Griess reagent system and sulfamic acid/H_2_SO_4_, respectively.

## 3. Results

### 3.1. Prokaryote Composition and Diversity

**Figure 2:**
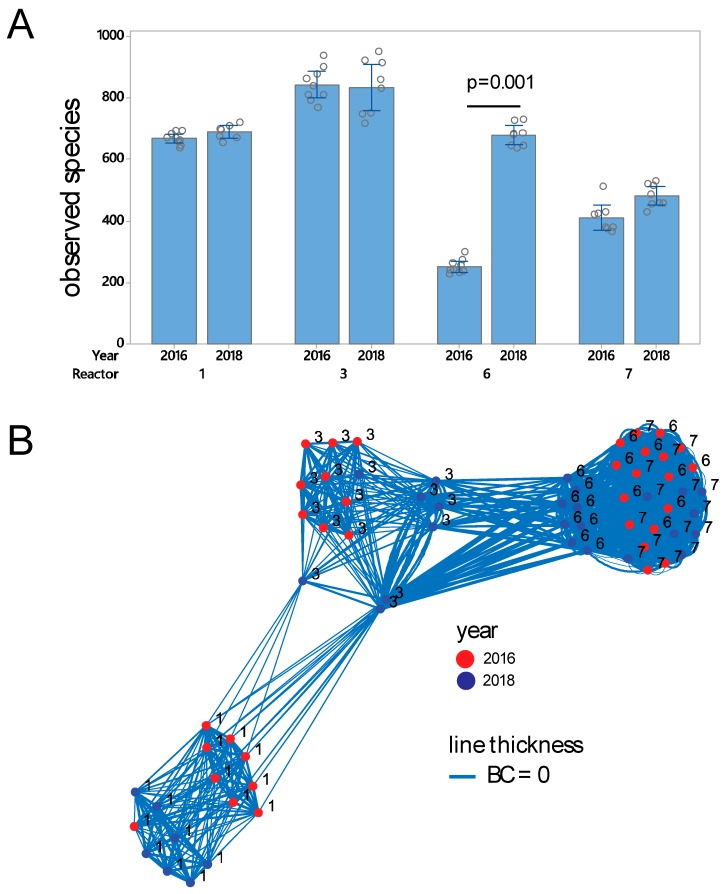
Prokaryote diversity measures. (**A**) α-diversity, as represented by observed species. Error bars represent standard deviations. (**B**) β-diversity, as represented by Bray–Curtis (BC) distances. Each sphere represents a biofilm. Bray–Curtis distances are represented with lines, with an inverse relation between distance and line thickness, with distances <0.5 being connected. The analyses were performed on the 66 samples with number of sequences above the rarefaction threshold of 10,000 sequences for the *16S rRNA* gene. The *p*-value was determined by the Kruskal–Wallis test.

In total, 2,885,789 sequences passed the quality filtering and 66 samples had >10,000 sequences. We found the highest species richness with over 800 OTUs in Reactor #3, whereas the lowest diversity was observed in Reactors #6 and #7. Reactor #6 showed a major difference in richness across years (*p* = 0.001, Kruskal–Wallis test), with a major increase in 2018 compared to 2016 (Figure 2A). Simpson and Shannon α-diversity indexes showed similar patterns for observed species (results not shown).

Bray–Curtis β-diversity analyses showed clear clustering according to reactor, with year representing sub-clusters (Figure 2B). β-diversity analyses revealed a tight clustering of samples from Reactors #6 and #7, with Reactor #3 showing diversity in-between Reactor #1, and Reactors #6 and #7 (Figure 2B). ASCA-ANOVA analyses confirmed the results from the β-diversity analyses, where reactor explained 84.6% of the variance in microbiota composition (*p* < 0.0001), and year only 1.5% (*p* < 0.0001).

Based on functional inference from taxonomic information in the MIDAS 2.0 database, Reactor #1 showed a high relative abundance of potential denitrifying bacteria (Supplementary Figure S2A). The highest level of nitrifiers was identified in Reactor #3 (Supplementary Figure S2B), with a dominance of *Nitrospira*. The level of nitrifiers was reduced in 2018 compared to 2016, with a complete absence of *Nitrosomonas* in 2018. Fermenters showed similar patterns as for denitrifiers (Supplementary Figure S2C), with *Flavobacterium* being the dominating genus. *Methylotenera* was overrepresented in Reactors #6 and #7 (Supplementary Figure S2D), where methanol was used as a carbon source. This genus, however, did not show known functional associations in the MIDAS 2.0 database [26].

### 3.2. Eukaryote Microbiota Composition and Diversity

**Figure 3:**
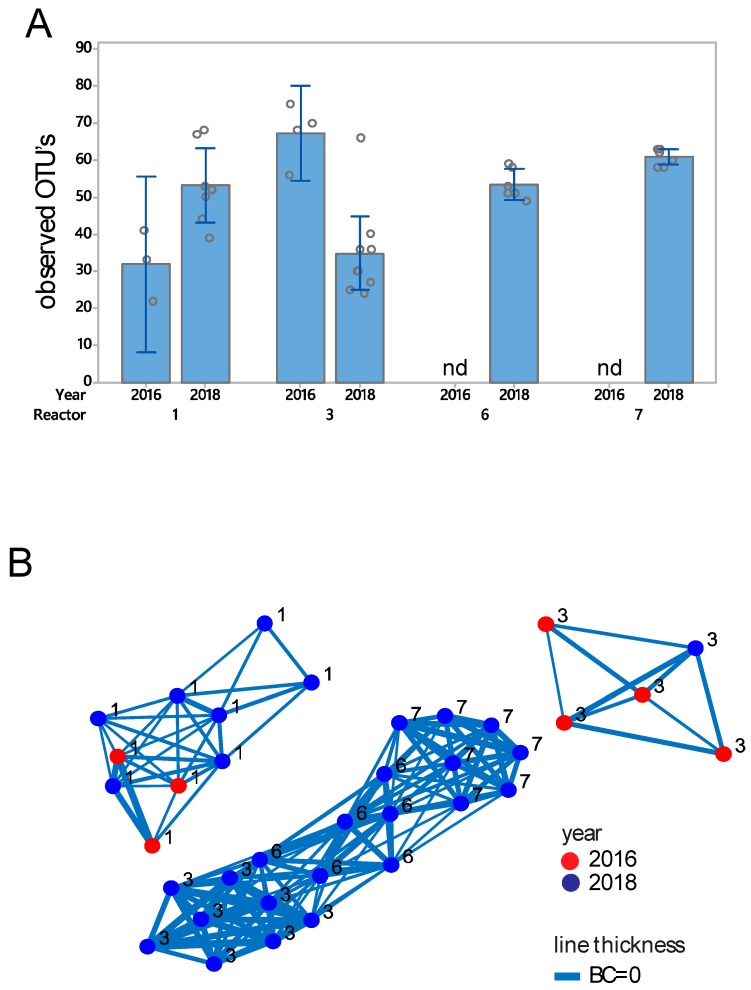
Eukaryote diversity measures. (**A**) α-diversity, as represented by observed species. Error bars represent standard deviations. (**B**) β-diversity, as represented by Bray–Curtis (BC) distances. Each sphere represents a biofilm. Bray–Curtis distances are represented with lines, with an inverse relation between distance and line thickness, with distances <0.5 being connected. The analyses were performed on the 36 samples with number of sequences above the rarefaction threshold of 10,000 sequences for the *18S rRNA* gene.

In total, 820,758 sequences passed quality filtering and 36 samples had >1000 sequences. The eukaryote species richness was found in the range of 20 to 80 OTUs, with no systematic difference across reactors (Figure 3A). Bray–Curtis β-diversity analyses showed a clustering of samples from Reactor #1 across 2016 and 2018, while the samples from Reactor #3 showed separate clustering in 2016 and 2018 (Figure 3B). ASCA-ANOVA analyses showed the highest explained variance for reactor, with 56.8% explained variance (*p* < 0.0001), while there was a considerable explained variance for year (9.4%, *p* < 0.0001).

With respect to composition, there was a dominance of a fungi classified as *Spizellomyces* for both 2016 and 2018 in Reactor #1 (Supplementary Figure S3E), while two OTUs classified as *Rhogostoma* (belonging to the Rhizaria supergroup) dominated in Reactor #3 (Supplementary Figure S3A,B). In Reactors #6 and #7, we identified high levels of *Adinetida* (kingdom animalia) in 2018, while the level of eukaryotes in these reactors was below detection in 2016 (Supplementary Figure S3D).

### 3.3. Functional and Strain Resolution Analyses in Reactors #6 and #7

We shotgun sequenced biofilms from Reactors #6 and #7 in order to better characterize genus *Methylotenera* in these reactors. We obtained a total of 25.6 million sequencing reads with a median length of 206 bp, covering a total of 5.1 billion bp. The pooled assembly yielded an N50 of 2701 bp, with the longest contig of 338,022 bp. The total contig length was 103 million bp. Details about the assembly are provided in Supplementary Table S1.

We first identified species/strain distribution across reactors and year in coverage analyses of pooled sequence assemblies. These analyses revealed that two biofilms from the same reactors had very similar compositions, with an average Spearman correlation for biobeads from the same reactor of 0.95 ± 0.05 (mean ± SD). The biobeads from Reactor #7 also clustered across the years, whereas the biofilms from Reactor #6 surprisingly clustered very differently in 2016 and 2018 (Supplementary Figure S4).

**Figure 4:**
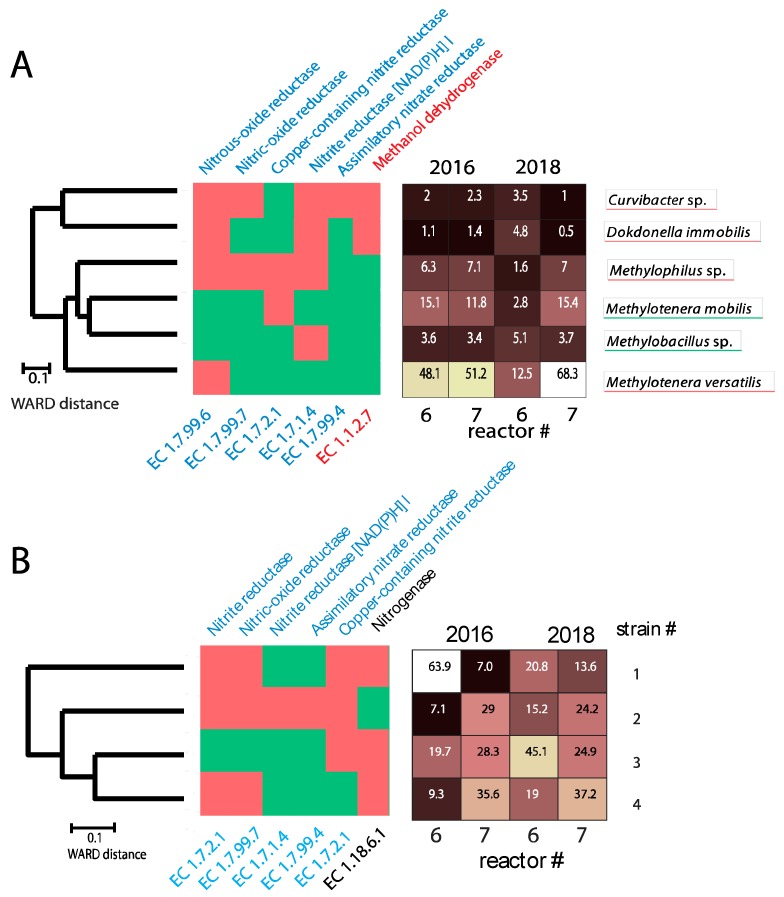
Gene content relatedness and distribution of species bins (**A**) and *M. versatilis* strains (**B**). The dendrograms represent the relatedness of the genomes/strains based on the total gene content with the color code indicating the presence (green) and the absence (red) of central genes encoding for searched enzymes. Genes important for denitrification are highlighted in blue, while the gene important for methanol utilization is marked in red, and the gene important for nitrogen fixation in black. The percentage of the species/strains within each reactor is also shown. The analyses are based on a total of 8 biofilms, two from each sampling point, with the mean values being presented.

Binning identified 14 metagenome bins for the assembled contigs (Table 1). Hierarchical clustering based on gene content in the genome bins with >70% completeness (*n* = 6) revealed two main clusters, being separated by the genes necessary for utilization of methanol, as indicated by the lack of genes with methanol dehydrogenase activity (Figure 4A).

The *M. versatilis* bin showed a size of more than 20 million pb, suggesting that bin represents a pan-genome, being composed of several strains. We therefore analyzed the contig coverage and distribution for this bin across years and reactors. This analysis revealed four main contig clusters (Supplementary Figure S5). Cluster #2 lacked all the genes for denitrification, while containing the machinery for nitrogen fixation and aerobic respiration, whereas the other clusters (Clusters #1, #3 and #4) contained different denitrification genes (Figure 4B).

In order to determine the global distribution of the identified *M. versatilis* strains, we searched all available shotgun metagenome raw sequencing reads against each of the four sequence clusters identified. These analyses revealed that strains related to those identified in Reactors #6 and #7 mainly reside in sediments, in particular, methane-enriched sediments (Supplementary Figure S6).

No proteins for nitrogen fixation were detected by proteome analyses, while high levels of proteins for methanol utilization (>5% of total identified proteins) were detected in both reactors. There was a higher expression of proteins for denitrification in Reactor #6 than in Reactor #7, whereas proteins involved in ribosome turnover were more abundant in Reactor #7 than in Reactor #6 (Supplementary Table S2).

### 3.4. Functional Characteristics of Biobead Performance

The overall performance for the nitrogen removal at the plant was slightly higher in 2016 compared to 2018. At the sampling time-points in 2016 and 2018, the total nitrogen in the inlet water were 36 and 37.2 mg/L, respectively, while the levels in the outlet water were 10.3 and 12.8 mg/L. This led to a nitrogen-removal efficiency of 71.4% in 2016, and 65.6% in 2018.

**Figure 5:**
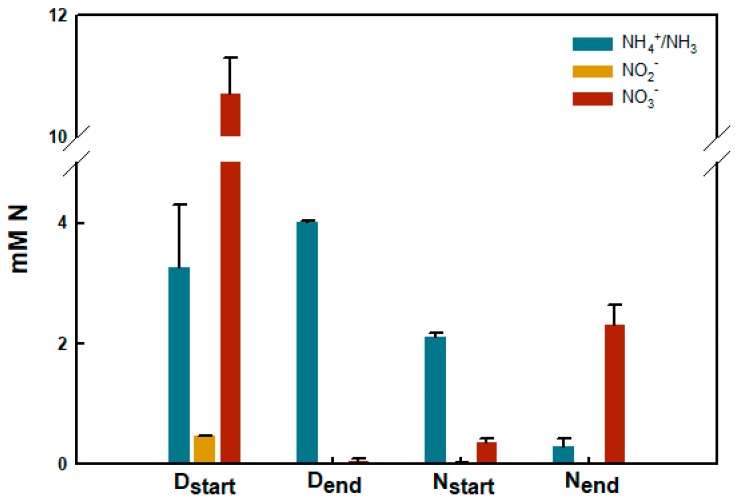
Concentration of NH_4_^+^/NH_3_, NO_2_^−^ and NO_3_^−^ at T = 0 and end of denitrification (D_start_/D_end_; Reactor #6 biobeads and liquid) and nitrification (N_start_/N_end_; Reactor #3 biobeads and liquid), measured by colorimetric assays. Samples were taken before the addition of biobeads (start) and after incubation under denitrifying and nitrifying conditions (end). The results from 2017 are presented.

An in-depth analysis was made of the functional characteristics of biobead performance for samples taken in 2017 and 2018 from Reactors #3 and #6. The results were largely identical; however, in 2018, N_2_O data were lost due to technical issues. Thus, the 2017 results are reported here. When biobeads were transferred to media with nitrate or ammonium under anoxic or oxic conditions, respectively, the available N-substrate was rapidly turned over. The microbiota on biobeads sampled from Reactor #3 oxidized approximately 87% of the available ammonium to stochiometric amounts of nitrate within 36 h of incubation (Figure 5). In order to quantify the formation of gaseous N-oxides during nitrification, biobeads were incubated in sealed serum vials under initial ambient atmosphere and headspace gases were monitored. Under nitrifying conditions (O_2_ in liquid >150 µM), the Reactor #3 community accumulated NO and N_2_O at nM concentrations. NO was kept below 7 nM in the liquid and the rate of N_2_O accumulation was 14.0 ± 0.80 nmol biobead^−1^ h^−1^. The Reactor #6 microbiota reduced all the available NO_3_^−^ to N_2_ within approximately 12 h of incubation and with minimal accumulation of the gaseous intermediates NO and N_2_O. N_2_O accumulated at increasing rates throughout the incubation (initial and maximum rate: 2.2 ± 0.8 and 14.5 ± 5.6 nmol biobead^−1^ h^−1^, respectively), but remained low (N_2_O_max_ = 2.2 ± 0.8 µmol vial^−1^). Likewise, NO was kept below 20 nM in the liquid (NO_max_ = 18.8 ± 2.3 nM) (Supplementary Figure S1 main panel). The total e^−^ flow to N-oxides was 10.5 ± 0.7 µmol e^−^ biobead^−1^ h^−1^ as long as NO_3_^−^ was available in the medium (Supplementary Figure S1 inserted panel).

## 4. Discussion

We found evidence of persistent and homogenous prokaryote microbiota composition over two years across biobeads in the same reactors. The distribution of the eukaryote microbiota, however, was less persistent, with no clear networks across years within the reactors. This may indicate that more stochastic and adaptive processes are involved in the eukaryote species assembly than for prokaryotes. The only persistent eukaryote colonizer in Reactor #1 was fungus related to *Spizellomyces,* which is known as a cosmopolitan degrader of organic material [33]. Although the nature of this fungus is unknown, it could potentially play an important role for utilizing cellulose as a carbon source in denitrification [34].

Reactor #3 showed a prokaryote composition apparently at an intermediary similarity compared to Reactors #1 and #6 based on Bray–Curtis. This may indicate downstream stochastic effects, as previously seen for water distribution systems [35], and for trickling filters [36]. However, the selective enrichment of nitrifiers in Reactor #3 is most likely due to niche selection for the process of nitrification within the biofilm, with the potential for comammox by *Nitrospira* [37]. The nitrification process was also observed experimentally. Reactor #6 showed the largest difference in prokaryote diversity and composition across years, with a marked decrease of *M. versatilis* in 2018 connected with an increase in species richness, indicating population dynamic processes. A factor that has not yet been considered in diversification is the effect of eukaryote–prokaryote interactions [8]. In 2018, eukaryotes showed a marked downstream effect with increasing α-diversity from Reactors #3 to #7, in addition to overlapping composition across reactors. Furthermore, we found a dominance of the *Rhogostoma* in both 2016 and 2018 for Reactor #3. This is a known eukaryotic predator [38]. In a microscopy examination (data not shown), we also confirmed predation by protozoa in Reactor #3. Thus, eukaryotes could also play an indirect role for prokaryote diversification [8,39].

Deterministic processes such as niche selection and cross feeding have been identified as important for maintaining prokaryote diversity both in simulated biofilm [40] and in batch cultures [41]. In a recent lab-scale experiment comparing methanol, ethanol and acetate as organic carbon sources in denitrification, major differences were detected for community composition, with *Methylotenera* dominating in the methanol-fed reactor [42]. This corresponds with our results where it appears that carbon source, rather than the level of oxygen or other electron acceptors (N-oxides), was the main determinant for shaping the microbiota composition in Reactors #6 and #7. Methanol amendment led to the selection of a stable population of *M. versatilis* strains, with the potential to exploit methanol in combination with oxygen and different redox states of nitrogen. Interestingly, there was an apparent lower efficiency of nitrogen removal in 2018 compared to 2016. This could potentially be related to the reduced Reactor #6 levels of *M. versatilis* in 2018. Furthermore, a reduced level of Reactor #3 nitrifiers could also interfere with the microbiota composition and nitrogen-removal efficiency.

Sequencing failed to reveal one or a few dominating full-fledged denitrifiers (capable of nitrate reduction to N_2_). Nevertheless, the system exhibited a high denitrification capacity. This may illustrate a modularity of the process in natural systems, where the individual reduction steps are likely conducted by a network of microbes carrying only parts of the denitrification apparatus. Moreover, the poor link between phylogeny and denitrification ability is in line with previous findings in bacteria isolated from soil and wastewater treatment systems [43,44].

Microbiota associated with spontaneously formed granular biofilms [45,46] appear to be temporarily and spatially less stable than biofilms on biocarriers [47]. Recently, biofilm thickness has been shown to affect the prokaryote microbiota composition and function in a deterministic manner [6,7]. This could partly be the reason why in granular systems, microbiota composition differs across granules in the same reactor [46]. The age of the biofilm could also be a contributing factor for the differences in diversity and stability between granular and biocarrier-based systems. We have recently shown that there was a 5-fold increase in phosphorous-accumulating organisms during a 1-year cycle for biofilm that was 7 months at the start of the experiment [9]. This indicates that the maturation of biofilm is a slow process, and that age can be a major factor determining composition. Since there are apparently a myriad of factors that could affect the niche composition for prokaryotes for granular-based wastewater treatment, it is difficult to separate stochastic and deterministic processes for such systems [48].

Although we did not identify signs of nitrogen fixation for *M. versatilis* in the wastewater treatment plant, the presence of nitrogen-fixing genes suggests that in the natural environment of this species, nitrogen fixation may be needed. Global distribution analyses indicated methane-enriched environments, such as sediments, as the natural habitat of *M. versatilis*. We also identified highly similar strains all over the globe, from arctic to desert environments. Both the widespread distribution and the methane/methanol association support strong environmental selection [49].

The eukaryotes identified have a diverse lifestyle, ranging from multicellular annelids to unicellular predators, and organic carbon-degrading fungi. Despite the diversity of lifestyles, eukaryotes have the common feature of being much larger than prokaryotes. The large size and lower numbers could therefore lead to lower persistence due to a larger influence of stochastic events for eukaryotes than for prokaryotes [50].

Taken together, our results show that the prokaryote biofilm microbiota exhibit taxonomic persistence over 2 years, while the eukaryote component was less stable. We therefore believe deterministic processes are more important for prokaryote than eukaryote biofilm assembly. This knowledge is of importance in understanding and controlling the processes.

Limitations of our study are that the sampling was only performed once each year, so we are not able to cover yearly fluctuations. Furthermore, the functional characterization of the microbiota was not conducted at the same time as determination of the microbiota composition, so we were not able to directly relate function and composition. The dentrification experiment was not run using methanol as a carbon source.

## Figures and Tables

**Table 1:** Metagenome bins identified by PATRIC database binning tool.

Genome Name	ID ^1^	Coarse Consist (%) ^2^	Completeness (%)	DNA Size (bp)	r6 ^3^ 2016	r7 ^3^ 2016	r6 ^3^ 2018	r7 ^3^ 2018
*Hyphomicrobium* sp.	113,574.7	74.8	54.94	2,070,006	0.9	0.7	0.9	0.4
*Hydrotalea flava*	714,549.25	88.4	15.74	274,213	0.2	0.1	0.1	0.04
*Methylophilus* sp.	1,112,274.5	82.9	71.41	2,555,358	6.3	7.1	1.6	7
*Methylotenera mobilis*	583,345.9	91.3	97.17	2,966,596	15.1	11.8	2.8	15.4
*Cupriavidus* sp.	367,825.5	86.5	12.52	511,195	0.5	0.4	0.4	0.3
*Methylotenera* sp.	1,506,585.5	87.5	19.59	811,227	4.4	3.2	0.9	4.1
*Flavihumibacter solisilvae*	1,349,421.6	83.6	14.17	434,301	0.1	0.1	0.2	0.1
*Polaromonas* sp.	296,591.29	75.7	49.85	1,455,211	0.9	0.9	1.6	0.5
*Rhodoferax ferrireducens*	338,969.31	71.5	44.59	2,352,732	1.5	1.7	2.5	0.9
*Luteibacter* sp.	1,798,239.6	75.7	35.08	1,107,355	0.5	0.4	0.9	0.3
*Curvibacter* sp.	1,797,748.7	80	83.62	2,770,618	2	2.3	3.5	1
*Methylobacillus* sp.	1,848,039.6	82.3	96.55	4,056,962	3.6	3.4	5.1	3.7
*Dokdonella immobilis*	578,942.18	80.5	96.55	8,908,583	1.1	1.4	4.8	0.5
*Methylotenera versatilis*	666,681.11	84	100	24,734,622	48.1	51.2	12.5	68.3

^1^ Species ID number for the closest match in the PATRIC database; ^2^ If a role in the genome is unexpected, or an expected role is missing, this is considered coarse inconsistency; ^3^ Numbers represent percentages of the respective genomes in the reactors.

## Supplementary Materials

The following are available online at https://www.mdpi.com/2073-4425/11/4/449/s1, Figure S1: Gas analyses in anoxic vials with 20 bio-beads from Reactor # 6 in 50 mL 9 medium supplemented with 10 mM KNO3, Figure S2: Functional assignments of the microbiota, Figure S3: Eukaryote composition of the microbiota, Figure S4: Correlation network based on shotgun sequencing coverage, Figure S5: Contig coverage across reactors and years for the *M. versatilis* bin, Figure S6: Coverage of the *M. versatilis* strains in metagenomes identified by SRA 40 searches, Table S1. Shotgun sequence assembly characteristics, Table S2. Proteome analyses of differentially expressed proteins.
