# Supplementary material for: Ecological Processes Affecting Long-Term Eukaryote and Prokaryote Biofilm Persistence in Nitrogen Removal from Sewage
Source: Genes (Basel). 2020 Apr 20;11(4):449. doi: 10.3390/genes11040449 (PMC7230490; doi:10.3390/genes11040449)
Supplement: Supplementary file 1 [file genes-11-00449-s001.pdf]

SUPPLEMENTARY INFORMATION

Suppl. Table 1. Shotgun sequence assembly characteristics

Parameters	Numbers
Number of contigs	42 069
Total length of contigs	102 829 072
Maximum contig length	338 022
N50[1]	2 701

[1] Minimum length of contig to cover 50% of the metagenome

Suppl. Table 2. Proteome analyses of differentially expressed proteins

Function	EC	r 6[1]	r7[1]	r7[1]
Nitrous oxide reductase	EC 1.7.99.6	1.29	0.67	0.39
Copper containing nitrite reductase	EC 1.7.2.1	2.42	1.16	1.49
Assimilatory nitrate reductase	EC 1.7.99.4	4.12	1.35	1.85
Methanol dehydrogenase	EC 1.1.2.7	10.62	6.40	7.12
Polyribonucleotide nucleotidyltransferase	EC 2.7.7.8	0.27	1.65	1.27

[1] Percentage of proteins identified

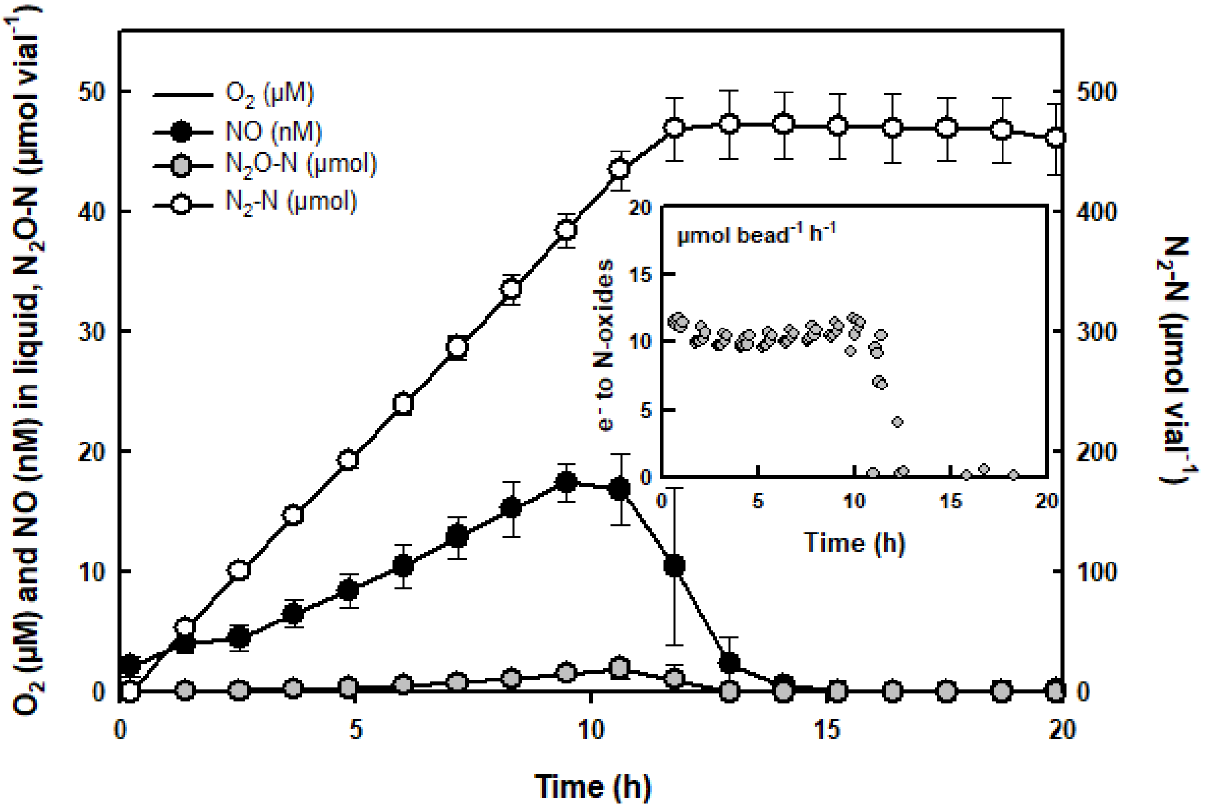

Suppl. Figure 1. Gas analyses in anoxic vials with 20 bio-beads from Reactor # 6 in 50 mL medium supplemented with 10 mM KNO_3. Main panel: Accumulation of NO, N_2O and N_2 during denitrification. Inserted panel: Total electron flow towards terminal electron acceptors (N-oxides). The plot is based on 6 replicates.

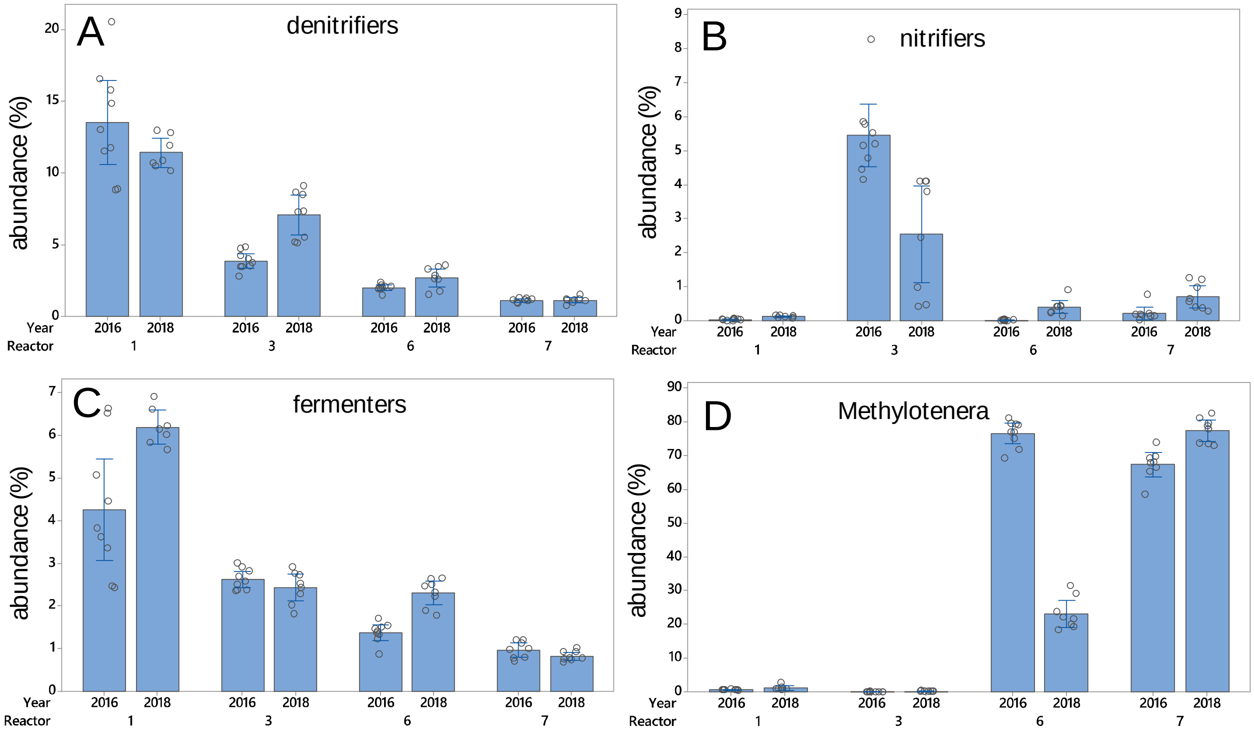

Suppl. Figure 2. Functional assignments of the microbiota. (A to C) Functional assignments were carried out using taxonomic matching with databases containing bacteria with a known function (MIDAS database). **(D)** Distribution of the overall most dominant genus *Methylotenera*, which was not classified into any functional group. The functional groups were defined from the MIDAS 2.0 database (McIlroy *et al.*, 2017). The analyses were performed on the 66 samples with number of sequences above the rarefaction treshold of 10 000 sequences for the 16S rRNA gene. Error bars represent satndard deviations.

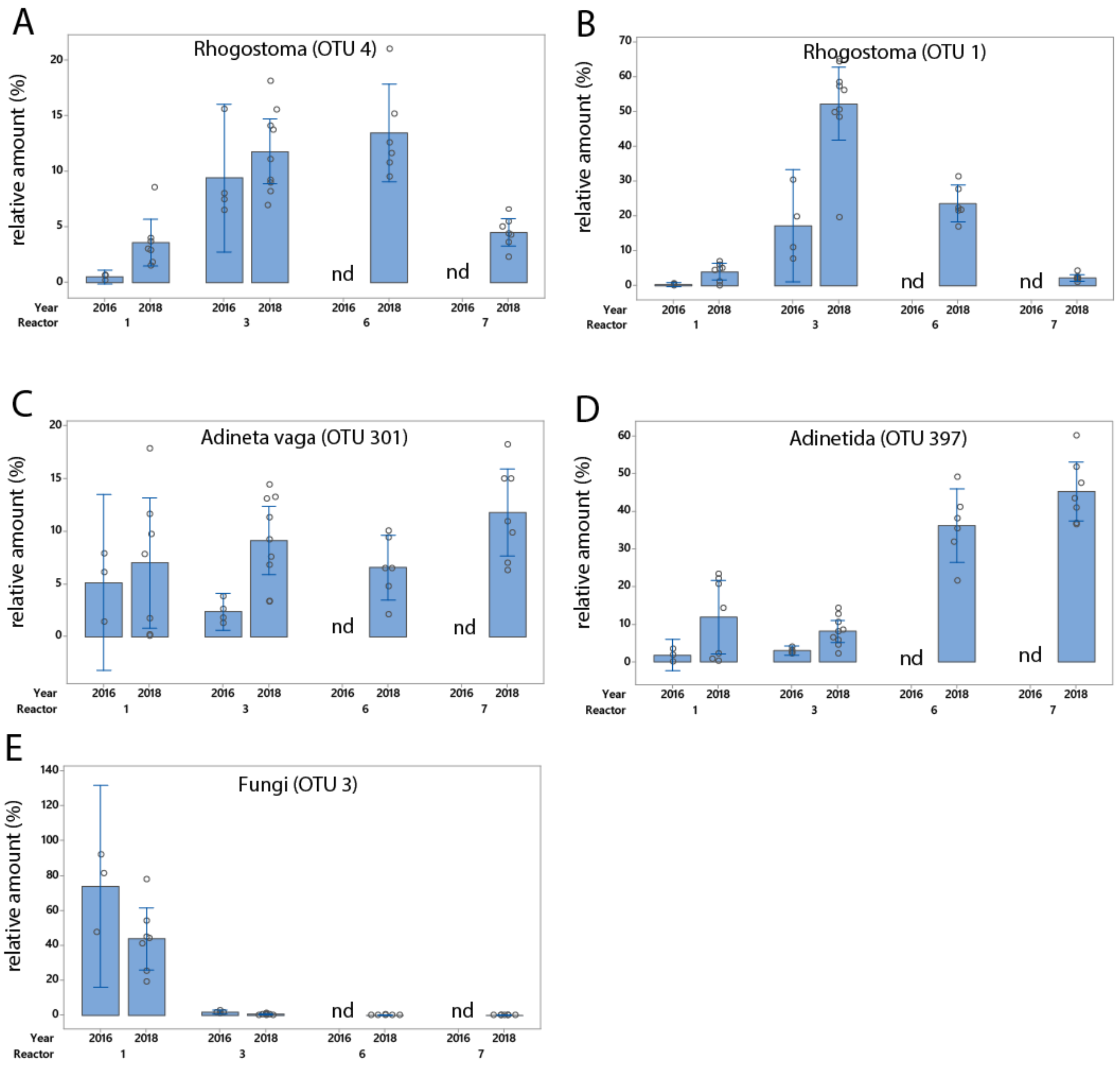

Suppl. Figure 3. Eukaryote composition of the microbiota. The distribution across reactors and years for the eukaryotes with an average abundance > 5% (panels A to E). The analyses were performed on the 36 samples with number of sequences above the rarefaction treshold of 1000 sequences for the 18S rRNA gene. Error bars represent standard deviations.

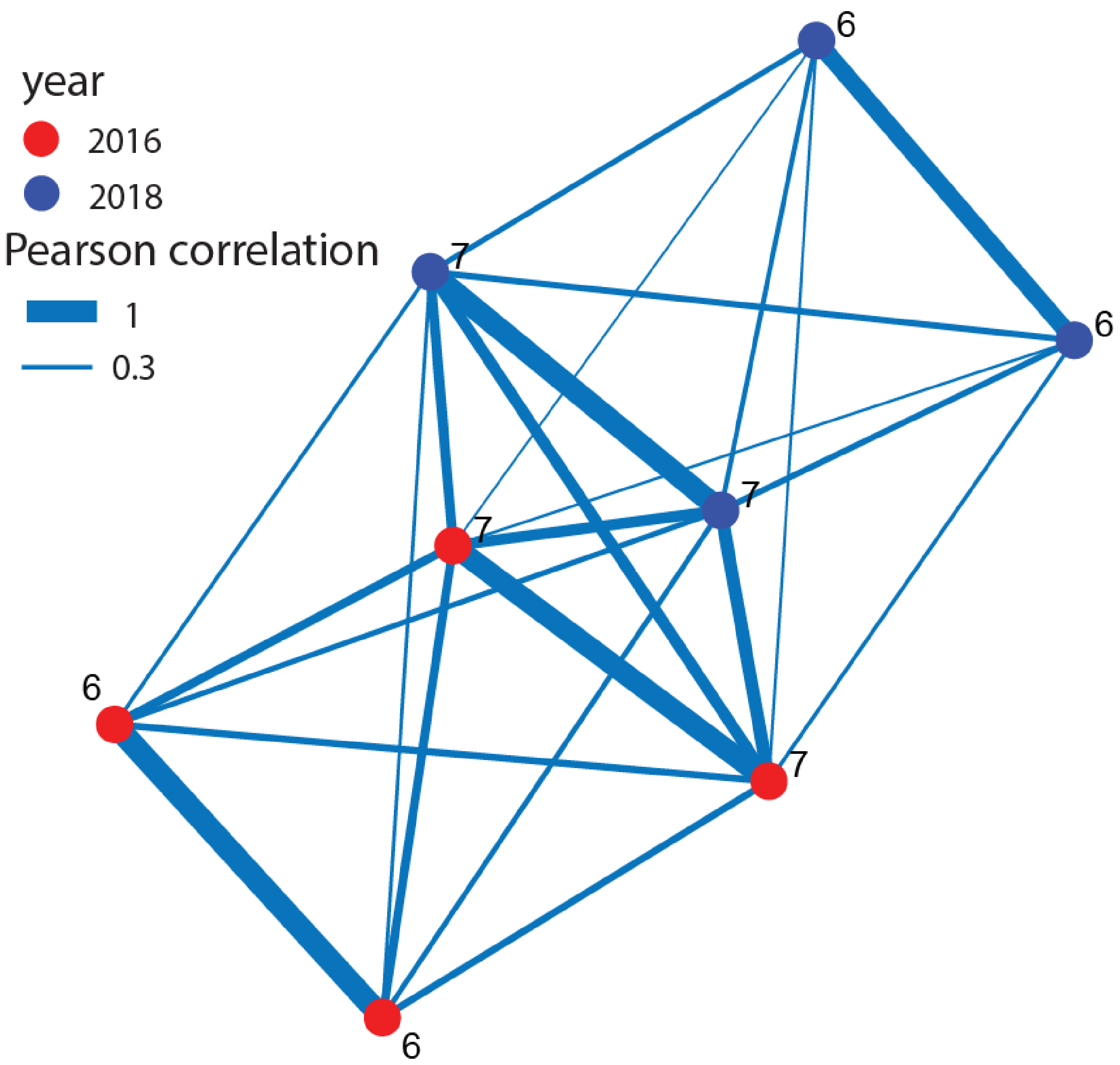

Suppl. Figure 4. Correlation network based on shotgun sequencing coverage. Nodes showing a Pearson correlation > 0.3 is connected, with the line thickening reflecting the correlation coefficient. The analyses are based on a total of 8 biofilms, two from each sampling point

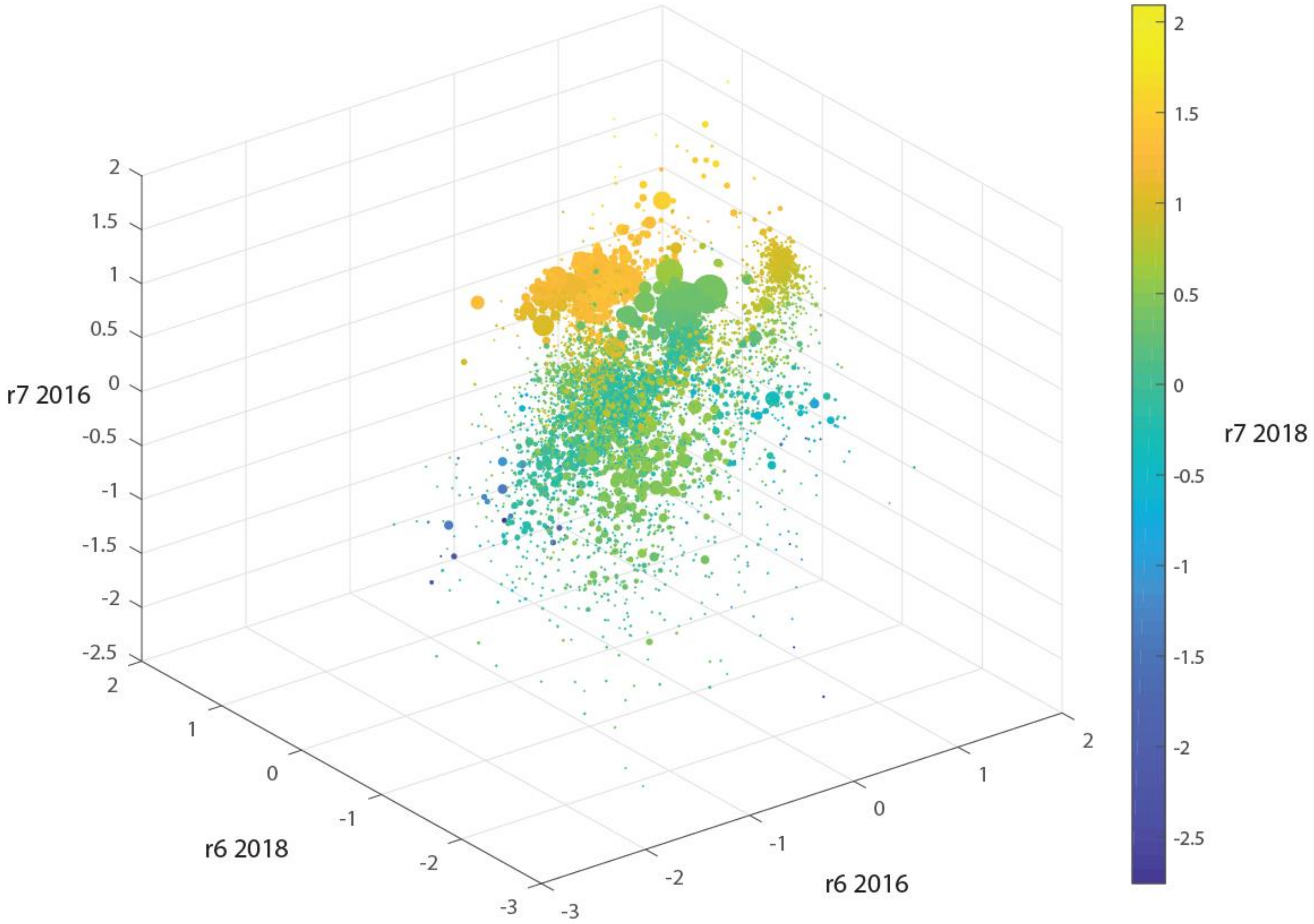

Suppl. Figure 5. Contig coverage across reactors and years for the *M. versatilis* bin. The coverage (log10) is illustrated in a 3D scatter plot, with the fourth dimension represented by a color code. The size of the spheres represent the length of the contigs, with the largest sphere representing a contig of 340 000 bp.

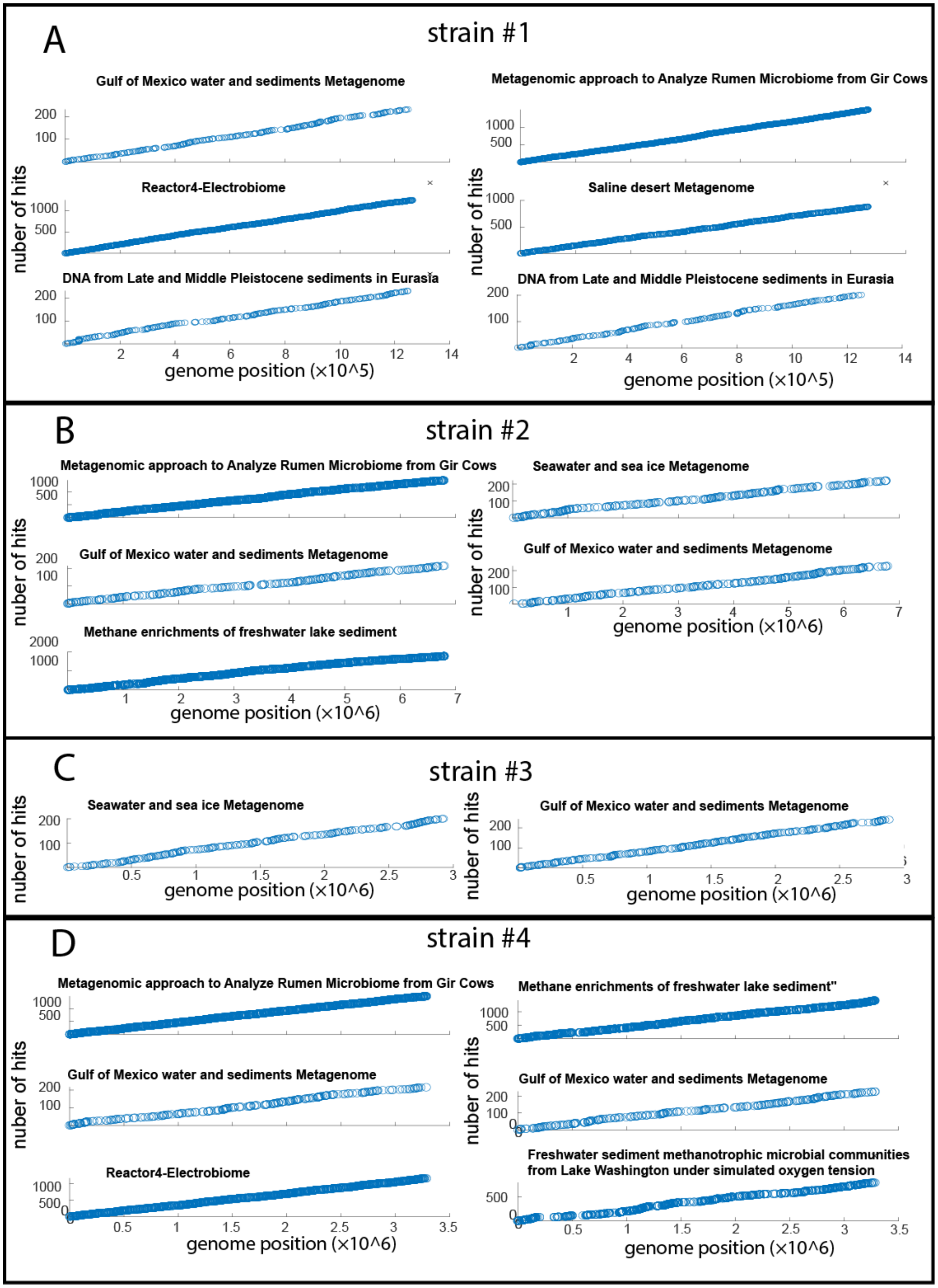

Suppl. Figure 6. Coverage of the *M. versatilis* strains in metagenomes identified by SRA searches. The hits are sorted along the respective genomes, with the titles indicating the origin of the metagenome.

43
